# Professional Assessment of the Impact of COVID-19 on Handling NET Patients

**DOI:** 10.3390/jcm9113633

**Published:** 2020-11-11

**Authors:** Sebastian Krug, Jakob Garbe, Senta König, Hanna Ungewiss, Patrick Michl, Anja Rinke, Jörg Schrader

**Affiliations:** 1 Department for Internal Medicine I, Martin-Luther University Halle/Wittenberg, Ernst-Grube-Straße 40, D 06120 Halle, Germany; jakob.garbe@uk-halle.de (J.G.); senta.koenig@gmail.com (S.K.); patrick.michl@uk-halle.de (P.M.); 2 IPSEN Pharma GmbH, Einsteinstraße 174, D 81677 Munich, Germany; hanna.ungewiss@ipsen.com; 3 Department of Gastroenterology and Endocrinology, University Hospital Marburg, Baldinger Strasse, D 35043 Marburg, Germany; sprengea@uni-marburg.de; 4 I. Medical Department-Gastroenterology and Hepatology, University Medical Center Hamburg-Eppendorf, Martinistrasse 52, 20246 Hamburg, Germany; jschrader@uke.de

**Keywords:** NET, neuroendocrine, COVID-19, SARS-COV-2, survey, treatment

## Abstract

The treatment and monitoring of patients with neuroendocrine tumors (NET) has been a major challenge during the COVID-19 pandemic. In a survey, we investigated the influence of COVID-19 on the care of NET patients in the German speaking countries Germany, Austria and Switzerland. The multidisciplinarity of all treating physicians in the outpatient and inpatient sector was reflected in our survey. Furthermore, we were able to present findings pertaining to the university and non-university medical care. Overall, only a minority of appointments were cancelled, mostly as a result of medical prioritization and less for fear of infection by patients. In the university sector, longer delays for diagnostic measures were observed in comparison to non-university care. During the COVID-19 crisis, NET patients rarely changed their current therapy, but the pandemic impacted the assessment of the different treatment modalities at risk of developing severe COVID-19 disease. This survey provides the first real-world data on the treatment of NET patients from the physicians’ perspective during the COVID-19 crisis. Despite delays in diagnostic procedures and outpatient appointments, only a minority of physicians foresee a major impact of COVID-19 on NET patient care.

## 1. Introduction

The outbreak of COVID-19 caused by Severe Acute Respiratory Syndrome Coronavirus 2 (SARS-COV-2) was declared as global health emergency by the world health organization (WHO) in January 2020. Until now COVID-19 has had detrimental effects on many aspects of human life, leading to both economic consequences such as high unemployment and recession, and heavy burdens on global health care systems [1,2].

The number of patients suffering from COVID-19 continues to rise worldwide, especially in some European countries like Spain and France. In Germany, the burden on the health care system is currently estimated to be relatively low in large parts of the country. However, with daily numbers of new cases again increasing in the last weeks, the burden could rapidly rise locally and then strain the public health systems, especially facilities for outpatient and inpatient medical care [3].

The COVID-19 pandemic has a significant impact on the care of patients with chronic diseases like cancer patients, who need frequent hospital admissions and visits. Cancer patients are at high risk for a severe course of COVID-19 due to several factors such as immunosuppressed state, higher age and comorbidities (e.g., chronic lung disease, diabetes, cardiovascular diseases). Therefore, in the COVID-19 era, oncologists should carefully weigh risks/benefits when planning cancer therapies and follow-up appointments [4].

ESMO guidelines classified cancer patients in two groups: patients off therapy (A) and patients under treatment (B). For all patients (A and B), it is mandatory to provide health education. For patients receiving active treatment (B), hospitals (physicians) should provide specific pathways in order to guarantee timing of treatment, modify regimen schedules to reduce number of clinic visits, favor phone or web-technology contacts. More intensive surveillance should be used during treatment for patients with lung cancer or those who received previous lung surgery, and for older patients or those patients with other comorbidities [5].

Furthermore, ESMO established a tiered approach for categorizing patients into three levels of priority (high, medium, and low) to receive active cancer treatment during the COVID-19 pandemic and developed “COVID-19 era recommendations” for the management of the main types of tumors [5]. However, for the care of patients with neuroendocrine tumors (NET) during the COVID-19 pandemic, there are no recommendations in either German or European guidelines [6,7].

Neuroendocrine neoplasias are rare tumor types. However, several studies have shown an enormous increase in the incidence in recent decades. The age-adjusted NET incidence has increased 6.4-fold from 1973 (1.09 per 100,000) to 2012 (6.98 per 100,000). The incidence and prevalence of NET are steadily rising, possibly owing to detection of early-stage disease and stage migration [1]. The diagnosis and treatment of NET patients requires a functioning multidisciplinary network in the outpatient and inpatient setting. Radiologists, gastroenterologists, endocrinologists, nuclear medicine specialists, and oncologists are involved in diagnostics. Some therapies such as somatostatin analogues (SSA), tyrosine kinase inhibitors (TKI) and chemotherapy are only performed on an outpatient basis, whereas peptide-mediated radionuclide therapy (PRRT) and locoregional therapy, for example, require inpatient monitoring. Therefore, the disease can be regarded as an indicator of a well-functioning hospital outpatient system and could consequently be affected very painfully by the COVID-19 pandemic.

There are several general recommendations from cancer societies and loco-regional reports [8,9,10], but hardly any published primary data or practical guidelines for diagnostics and therapies of NET in the era of COVID-19. The first NET-specific guideline was published 4 months after the pandemic had started and was not yet available at the time this survey was conducted [11]. An Italian survey showed that the spread of the COVID-19 pandemic had a significant impact on the management of NET patients, reducing the number of newly diagnosed patients, deferring a significant proportion of surgical procedures, and delaying the beginning of PRRT [12].

In this study we assessed the impact of COVID-19 on current NET patient care in Germany. We conducted a survey to investigate how strongly further diagnostic and therapeutic procedures of NET patients in outpatient and inpatient settings were affected.

## 2. Methods

Ethical approval was obtained from the local ethical review committee of the Martin-Luther-University Halle/Wittenberg (number: 2020-093; June 2020). A total of 16 questions were developed, including four general questions about characteristics of physicians and facilities and 12 specific questions about the influence of COVID-19 on the management of NET patients. Either several answers of a given choice, only one answer (multiple choice) or yes/no answers were possible. In some cases, free text was possible to specify the answer.

The survey was performed between 1 July and 31 July in the following three German-speaking countries: Austria, Switzerland and Germany. The survey was open to physicians who treat NET patients in inpatient and outpatient settings, regardless of the disease stage and whether they received current therapy or follow-up care. Although there was no restriction regarding the inclusion of COVID-19-positive patients, it can be assumed that the responding physicians only saw COVID-19-negative patients, because COVID-19-positive patients were treated separately in the inpatient area depending on the care structure and were not treated routinely in the outpatient area in order to minimize the risk of transmission. The questionnaire and was distributed via the German NET-Registry and via personal contacting. The full survey is provided in the Supplemental Material.

The software LimeSurvey (LimeSurvey GmbH, Hamburg, Germany) was used to conduct the survey online. Descriptive statistics were calculated using Office Excel 2016 (Microsoft Corporation, Redmond, WA, USA) and GraphPad Prism 5 (GraphPad Software, San Diego, CA, USA).

## 3. Results

### 3.1. Characteristics of Physicians and Facilities

**Figure 1:**
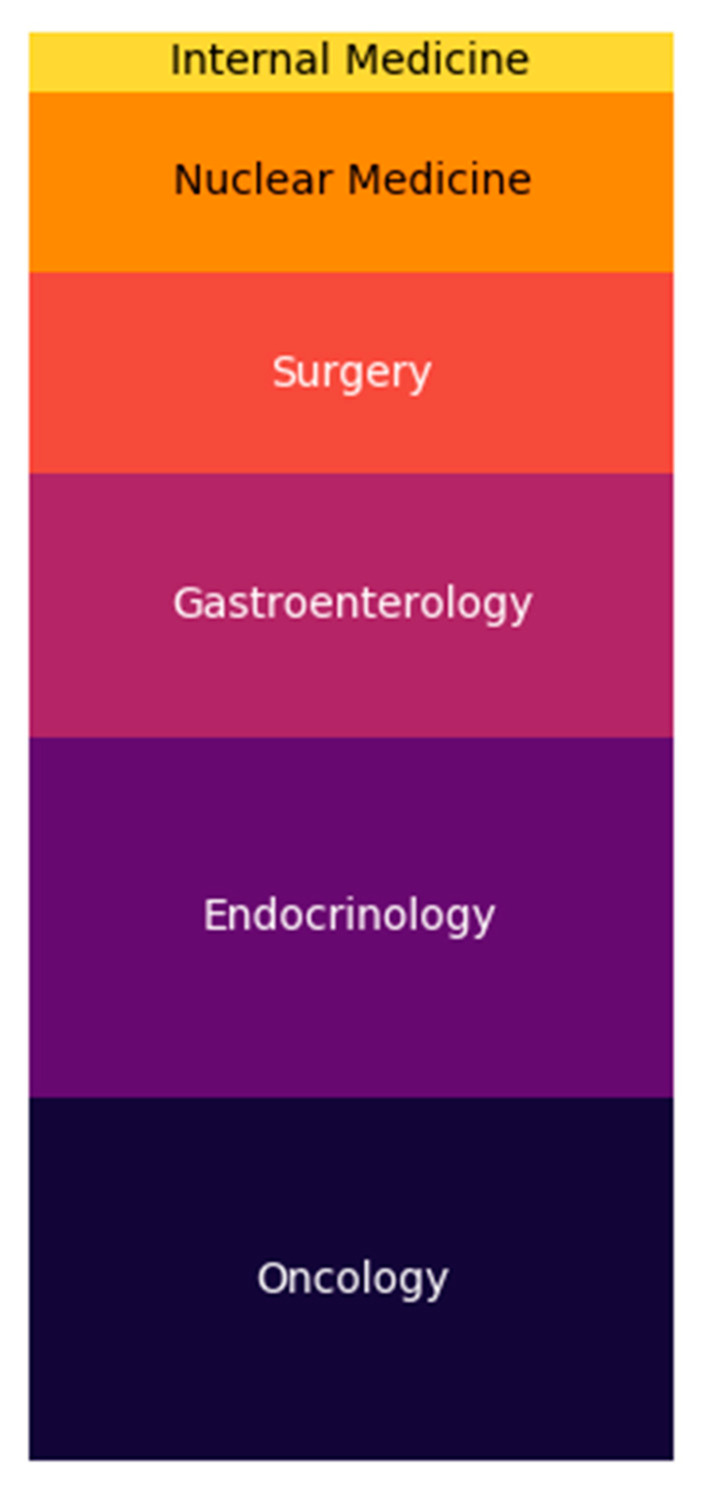
Survey responders’ discipline.

A total of 92 participants took part in the survey and 77.2% (*n* = 71) of the participants answered all questions completely. Most physicians worked in a university hospital (55%, *n* = 39), followed by non-university hospitals (30%, *n* = 21) and specialist practices (practice for internal medicine offering medical oncological treatment; 15%, *n* = 11). The question of clinical specialization was answered as follows: oncology (25%, *n* = 18), endocrinology (25%, *n* = 18), gastroenterology (18%, *n* = 13), surgery (14%, *n* = 10), nuclear medicine (13%, *n* = 9), internists (4%, *n* = 3) (Figure 1). In most facilities, more than 25 NET patients are treated annually (65%, *n* = 46). Typically, physicians spend 5–20% of their time treating NET patients. Only 6 participants spend more than 40% of their time on the care of NET patients.

### 3.2. Influence of COVID-19 on Outpatient and Inpatient Care

**Figure 2:**
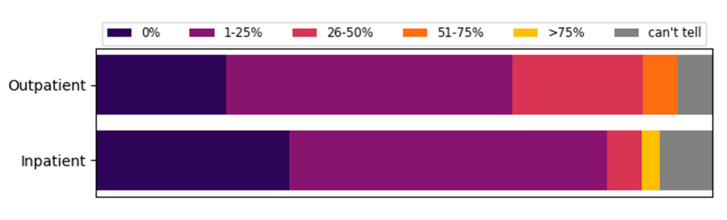
Fractions of cancelled outpatient appointments and inpatient admissions of NET patients due to the COVID-19 pandemic as estimated by NET professionals. Outpatient cancellations: 0% (*n* = 15/22%), 1–25% (*n* = 33/49%), 26–50% (*n* = 15/22%), 51–75% (*n* = 4/6%), >75% (*n* = 0). Inpatient cancellations: 0% (*n* = 22/34%), 1–25% (*n* = 36/56%), 26–50% (*n* = 4/6%), 51–75% (*n* = 0), >75% (*n* = 2/3%).

Based on the physician characteristics, responses from university centers and non-university centers were evaluated separately (see Supplementary Materials Table S1). The first four questions of the specific part examined the influence of COVID-19 on outpatient and inpatient care and how often appointments had to be cancelled due to the pandemic. In most cases (cancellations less than 25%), outpatient and inpatient appointments were realized (71%, *n* = 48 and 90%, *n* = 58 respectively). We found that 55% (*n* = 39) and 62% (*n* = 44) of the appointments for the treatment of NET patients were never or rarely postponed or cancelled for outpatient and inpatient treatment, respectively. In the inpatient sector, 9% (*n* = 6) of the participants stated that more than 25% of the consultations were cancelled, while the corresponding number in the outpatient sector was significantly higher (28%, *n* = 19) (Figure 2). The influence of COVID-19 on care and cancellations tended to be higher in the university than in the non-university sector, e.g., 49% of physicians in universities vs. 28% of physicians in non-university sectors stated that COVID-19 has a strong or very strong influence on outpatient care.

**Figure 3:**
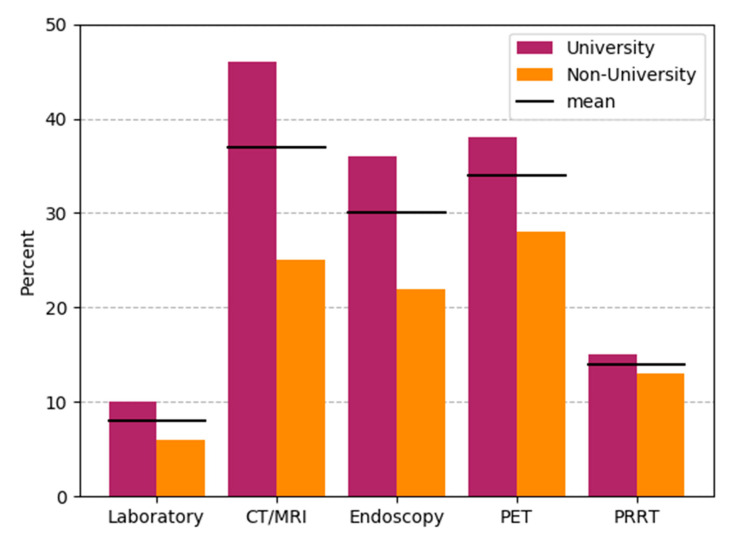
Delays in diagnostics and therapy of NET patients. Bars are drawn separately for university hospitals (*n* = 39) and non-university-based care such as non-university hospitals and private practices (*n* = 32). Black horizontal bars indicate results for combined responses (*n* = 71). PRRT: Peptide Receptor-mediated Radionuclide Therapy.

Patients with NET require a wide range of laboratory, endoscopic and radiological/nuclear medical procedures. Approximately 33% of the participants stated that there were delays in radiological/nuclear medical (CT/MRI 37%, *n* = 26), endoscopic workups (30%, *n* = 21) and with FDG/Ga68-PET/CT (34%, *n* = 24), with more extensive delays in the university sector (46% vs. 25%, 36% vs. 22% and 38% vs. 28%, see question C6, Figure 3). Minor delays occurred in laboratory medicine and PRRT. In 19 participants (27%), it was stated that there were no delays in general.

Whether the COVID-19 situation has a long-term effect on referrals/admissions to NET patients was negated in 57% (*n* = 40). Only 13% (*n* = 9) believed that this will have a significant impact in the future.

### 3.3. Reasons for Cancellation/Postponing of Appointments

When asked about the reasons for postponing a consultation, medical priority ranked higher (85%, *n* = 60) than the patient’s preference (44%, *n* = 31). Twenty percent (*n* = 14) of the participants reported that more than 20% of all cases were cancelled due to the patients’ fear of an infection, while 80% (*n* = 56) stated that less than 20% of the cases were cancelled due to this reason. We found that 47% of physicians were asked frequently/very frequently by patients about their personal risk of disease. In the university setting the demand was higher than in the non-university setting (59% vs. 37%).

### 3.4. Risk for a Severe Course of COVID-19 and Recommendation of COVID-19 Tests

**Figure 4:**
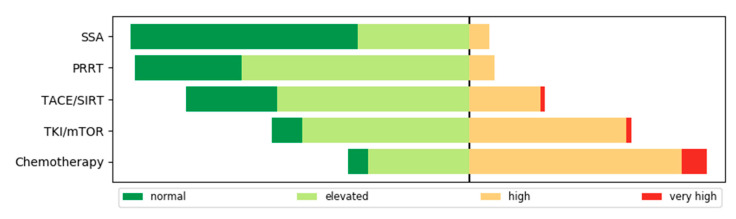
Risk of severe courses of COVID-19 infection under five NET therapy regimens according to physician rating. SSA [63%, 31%, 6%, 0%], PRRT [30%, 63%, 7%, 0%], TKI/mTOR: Everolimus and Sunitinib [8%, 46%, 44%, 1%), TACE/SIRT: Transcatheter arterial chemoembolization and selective internal radiation therapy [25%, 54%, 20%, 1%] and chemotherapy [6%, 28%, 59%, 9%]. For reasons of clarity, the bars have been centered between elevated and high risk.

One of the main objectives of this survey was to evaluate the different existing therapies for NET patients according to their risk for a severe course of COVID-19 infection. As shown in Figure 4, the risk assessment revealed SSA and PRRT with low risk, whereas targeted therapies and chemotherapy were attributed a higher risk. The loco-regional therapeutic procedures appeared in-between. Non-university physicians noted a higher elevated risk regarding SSA and PRRT (47% and 75%) than physicians in universities (18% and 54%), in particular with respect to loco-regional therapy procedures (increased risk estimated 31% vs. 10% in the university sector) (supplementary Figure S1). When questioned whether an ongoing therapy had to be postponed, changed or discontinued, 76% (*n* = 54) of the respondents answered “no”. In 24% (*n* = 17) of cases this was rarely carried out.

Overall, a COVID-19 test was recommended in 62% of NET patients; more often in non-university than in university settings (78% vs. 49%).

## 4. Discussion

Care of patients with neuroendocrine tumors demands an interdisciplinary approach with regular in- and outpatient appointments. Although the course of the disease is frequently slower than in other tumor entities, there is still a high need for care in patients with advanced disease. In our survey we aimed to assess the impact of the SARS-COV-2 pandemic on patient care for neuroendocrine tumors from the perspective of the treating physicians. Although the pandemic influenced appointments, diagnostic procedures and preferences for treatment, only a minority of participating physicians were worried about a significant impact in the future.

During the lockdown phase of the SARS-COV-2 pandemic in Austria, Switzerland and Germany, most hospitals were faced with strict regulations regarding patient contacts. This was reflected in our survey as most participants experienced a strong impact on outpatient appointments. Interestingly, this effect was much stronger in the university setting than in non-university hospitals and private practice. Current ESMO guidelines emphasize the evaluation of phone consultations and video contacts with patients to avoid hospital or outpatient clinic visits [5]. Whether this practice has also been implemented by the participating physicians is not known. Still, cancellation for outpatient or inpatient appointments was less than 25% in the majority of participants. This is in contrast to a recently published survey from Italy, where 26% of appointments were delayed and newly diagnosed NET was reduced by 77% [12], possibly reflecting the much stronger impact of COVID-19 on Italy than on German speaking European countries. Although the major reason for cancellations were due to triage for medical priority, up to 20% of physicians reported relevant cancellations by patients due to fear of a SARS-COV-2 transmission in the clinic. Reports of hospital outbreaks of SARS-COV-2 infections have likely influenced this fear of infection.

As NET is a rare disease and requires a multi-professional team for appropriate care, most patients are treated in specialized centers within university hospitals. In the lockdown phase, these specialized hospitals were strictly regulated by governmental action to prioritize activities towards care for patients with COVID-19. Thus, it is not unexpected that physicians in a university setting report delays in diagnostic procedures more frequently than physicians in non-university hospitals or private practice. Although imaging procedures were delayed by up to 50% in university hospitals, no major delays for PRRT treatments were reported. This is in stark contrast to the data from the Italian survey, who reported delays in initiating PRRT treatment in up to 50% of patients [12].

When asked about patients worries related to COVID-19 and requests for assessment on patient’s personal risks, physicians reported a high demand for information. As most NET patients either have an ongoing treatment for metastatic disease or have had a major operation in the past, these patients might fall into categories of increased risk for a severe disease course of COVID-19 [13]. Furthermore, the majority of patients belong to the age group above 60 years, incurring an additional risk factor for a severe disease course. Although data on previous surgery and risk of severe COVID-19 disease are still lacking, splenectomy—as often performed during surgery for pancreatic neuroendocrine tumors—might be associated with a complicated course of SARS-COV-2 infection in analogy to influenza infection [14]. So far, no survey has shown an increased risk for patients after major abdominal surgery or splenectomy; thus, this group of NET patients might not be at an increased risk. As this information has not been available in the first months of the pandemic, counseling by treating physicians has been difficult and patients were left with a high degree of uncertainty. The high demand for information from the patients reflects this uncertainty and the need for patient-centered information on the implications of SARS-COV-2 for NET patients.

The most important point of the survey was to assess how the SARS-COV-2 pandemic would influence choice and decisions for treatment in NET patients. Risk assessments of treatments have been published by medical societies for other tumor entities [4]. Only recently, some general recommendations for NET patients have been published by an expert group [15]. In our survey, most physicians did not attribute a high risk of severe COVID-19 disease to patients treated with SSA. This is in line with the recommendations by Casey et al. and reflects practice guidance in breast cancer patients receiving anti-hormonal treatments [16]. Interestingly, the same low risk has been attributed to PRRT treatment, which might be in contrast to recommendations to restrict extensive radiotherapy during the SARS-COV-2 pandemic [5]. Bodei et al. recently published an opinion paper reflecting possible risks of PRRT in NET patients [17]. Despite the lack of clinical data on PRRT and risk of severe COVID-19, they envision PRRT to be a safe treatment option during the pandemic. Although local ablative treatments like TACE or SIRT do not influence the immune system directly or cause myelosuppression, there was a substantial fraction of physicians who attributed a high risk of severe COVID-19 to this treatment. Interestingly, there was a relevant difference between university (low risk attribution)- and non-university (high risk attribution)-based physicians, possibly reflecting the higher familiarity with these procedures in university settings. Molecular targeted therapies have been attributed to a higher risk by university-based physicians than by non-university physicians, again potentially reflecting the more familiarity with molecular targeted therapies as used in other tumor entities by oncologists in private practice. Unfortunately, we did not differentiate between sunitinib and everolimus in our survey, but we suspect that most risk is attributed to everolimus rather than to sunitinib. Indeed, a survey among oncologists who treat patients with renal cell cancer report a preference for TKI during the SARS-COV-2 pandemic [18]. As everolimus can both cause pneumonitis and is an effective immunosuppressant, this likely caused the fear in using this treatment during the pandemic. Nevertheless, there are opinion reports speculating on a potential benefit of mTOR inhibition in suppressing the overwhelming immune response in severe COVID-19 disease [19]. The highest risk for a severe course of SARS-COV-2 infection was attributed to systemic chemotherapy treatment. Given the potential myelosuppression and immunosuppression caused by chemotherapy, this has been expected. Indeed, retrospective series report a higher mortality rate of COVID-19 patients when on active cancer treatment receiving chemotherapy [13,20]. In contrast, other register data did not find an excess mortality for cancer patients on active treatment [21,22]. As systemic chemotherapy is only used in pancreatic NET patients, where several alternative treatments are available, it might be justified to delay initiation of systemic chemotherapy in NET patients. In light of the second SARS-COV-2 wave currently sweeping across Europe, consideration may be given to particularly treat patients with a first diagnosis in an advanced stage of the disease primarily with PRRT in addition to SSA and also to critically examine whether ongoing therapy should be switched to therapies that have been assessed as safe in the survey. For instance, SSA and PRRT were classified as safe, whereas the other therapies, such as TKI, chemotherapy, and locoregional therapies were regarded as more critical because SSA and PRRT require less monitoring and fewer follow-up visits compared to the other therapies.

One limitation of this study is the purely observational character relying on the participation of contacted physicians. Thus, this survey does not include all centers treating NET patients nor all specialties involved in the care of NET patients. Centers of Excellence (COEs) were not specified explicitly. Nevertheless, we received responses from six different groups of physicians involved in the care of NET patients. Not unexpected, most participants in this survey were oncologists, endocrinologists and gastroenterologists providing outpatients care for NET patients. The number of participants does not reflect the number of participating hospitals, because potentially multiple colleagues from one and different disciplines per hospital could take part in the survey. The survey questions were compiled very succinctly in order to increase the response rate. Unfortunately, we did not include questions on specific measures taken by different physicians to adjust medical care to the crisis. Thus, we cannot report on the use of video-consultations, phone consultations or community-based patient care as recommended by oncological societies. The survey did not distinguish between advanced high-grade neuroendocrine neoplasms (NEN) and advanced low grade NEN as physicians were addressed who treat NET patients in general, regardless of the disease stage. Nor did the survey distinguish between COVID-19-positive and COVID-19-negative patients. It can be assumed, however, that the survey only reflects the practice of COVID-19-negative patients. COVID-19-positive patients were treated separately in the inpatient area depending on the care structure and were not treated routinely in the outpatient area in order to minimize the risk of transmission.

## 5. Conclusions

This survey provides the first real-world insight into the physician’s attitude towards the SARS-COV-2 pandemic and the experience and consequences for the treatment of patients with neuroendocrine tumor disease. Although some delays in diagnostic procedures were experienced in the lockdown phase of the pandemic, only a minority of physicians report a major impact of the pandemic on care for NET patients in Germany, Austria, and Switzerland.

## Supplementary Materials

The following are available online at https://www.mdpi.com/2077-0383/9/11/3633/s1, Table S1: Questionnaire; Figure S1: Risk of severe courses of COVID-19 infection under five NET therapy regimens according Table 79.
